# Supplementary material for: Circulating tumor DNA refines risk stratification of neoadjuvant therapy-resistant breast tumors
Source: Nat Commun. 2025 Dec 9;16:9945. doi: 10.1038/s41467-025-65432-5 (PMC12690079; doi:10.1038/s41467-025-65432-5)
Supplement: Supplementary file 1 — Supplementary Information [file 41467_2025_65432_MOESM1_ESM.pdf]

SUPPLEMENTARY INFORMATION

Circulating tumor DNA refines risk stratification of neoadjuvant therapy-resistant breast tumors

Mark Jesus M. Magbanua[1], Nayelis A. Manon[1], Denise M. Wolf[1], Samuel Rivero-Hinojosa[2], Ziad Ahmed[1], Rosalyn W. Sayaman[1], Antony Tin[2], Derrick Renner[2], Ekaterina Kalashnikova[2], Lamorna Brown-Swigart[1], Gillian L. Hirst[3], Christina Yau[3], Wen Li[4], Claudine Isaacs[5], Rebecca A. Shatsky[6], Amy Clark[7], Alexandra Zimmer[8], Amy L. Delson[9], Angel Rodriguez[2], Minetta C. Liu[2], Paula R. Pohlmann[10], Laura J. Esserman[3], Hope S. Rugo[11], Angela DeMichele[7], and Laura J. van 't Veer[1]

[1] Department of Laboratory Medicine, University of California San Francisco, San Francisco, California, USA

[2] Natera, Inc., Austin, Texas, USA

[3] Department of Surgery, University of California San Francisco, San Francisco, California, USA

[4] Department of Radiology, University of California San Francisco, San Francisco, California, USA

[5] Lombardi Comprehensive Cancer Center, Georgetown University Medical Center, Washington, DC, USA

[6] Department of Medicine, University of California San Diego, La Jolla, California, USA

[7] Division of Hematology/Oncology, University of Pennsylvania, Philadelphia, Pennsylvania, USA

[8] Division of Hematology/Oncology, Oregon Health and Science University, Portland, Oregon, USA

[9] Breast Science Advocacy Core, University of California San Francisco, San Francisco, California, USA

[10] Department of Breast Medical Oncology, University of Texas MD Anderson Cancer Center, Houston, Texas, USA

[11] Division of Hematology/Oncology, University of California San Francisco, San Francisco, California, USA

SUPPLEMENTARY FIGURES

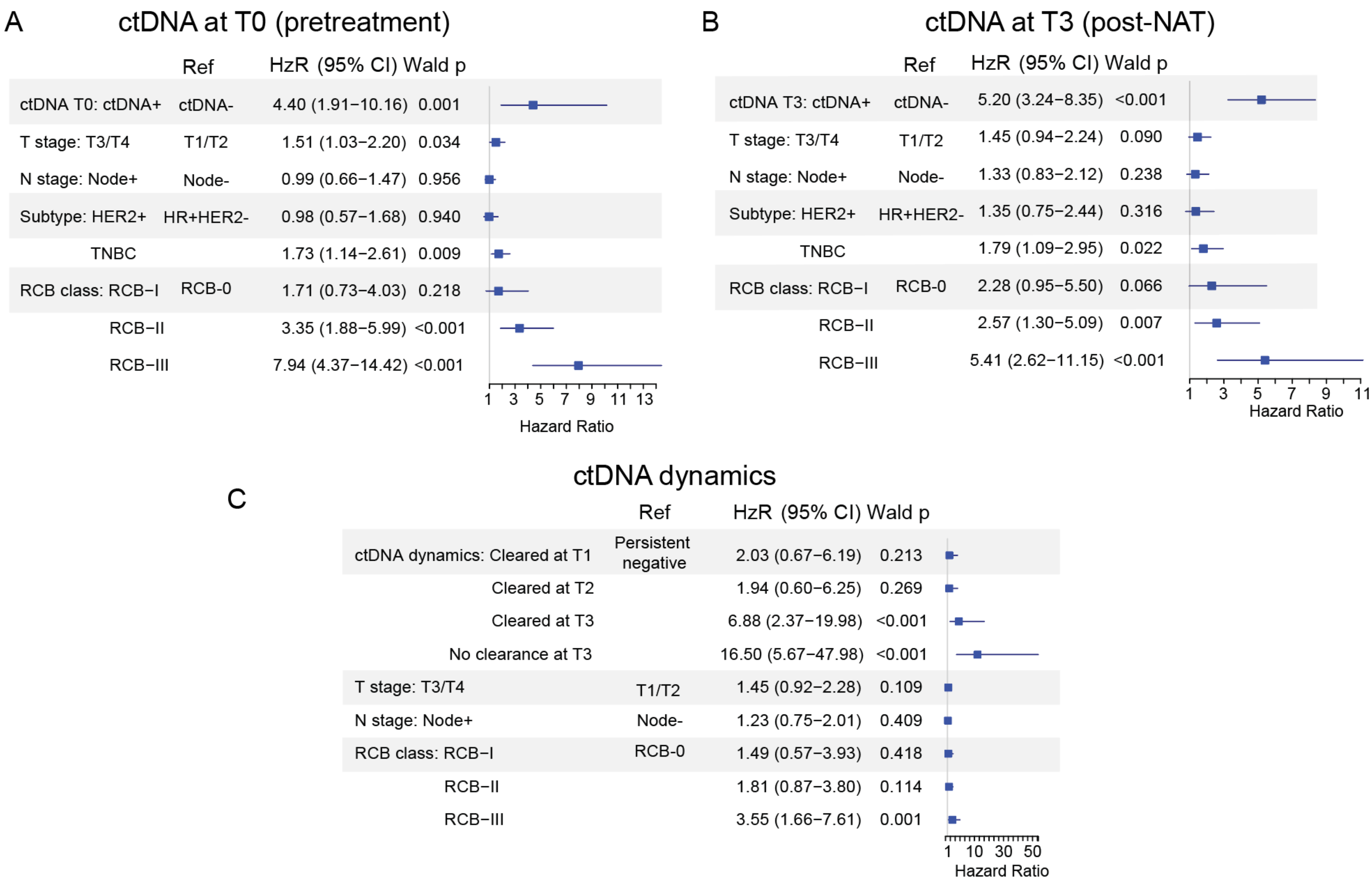

Figure S1. Circulating tumor DNA (ctDNA) is an independent predictor of distant recurrence-free survival (DRFS) in patients with high-risk early-stage breast cancer who received neoadjuvant therapy (NAT). Multivariable Cox regression analyses included clinicopathologic variables that were statistically significant in univariable analyses (**Table S2**) to evaluate the prognostic value of **A.** ctDNA positivity at pretreatment (T0), **B.** post-NAT before surgery (T3), as well as **C.** ctDNA dynamics (timing of ctDNA clearance). Patients were grouped by ctDNA dynamics: persistent ctDNA-negative, ctDNA cleared at T1 (week 3), T2 (week 12), or T3 (post-NAT before surgery), or no ctDNA clearance post-NAT before surgery. Cox regression analysis was used to estimate hazard ratios (HzR) and 95% confidence intervals (CI). The p-values were calculated using the Wald test.

A ctDNA status at T0 and RCB-II/II

Pairwise log-rank tests

	RCB-II ctDNA+	RCB-II ctDNA-	RCB-III ctDNA+
RCB-II ctDNA-	0.004	NA	NA
RCB-III ctDNA+	<0.001	<0.001	NA
RCB-III ctDNA-	0.399	0.185	0.013

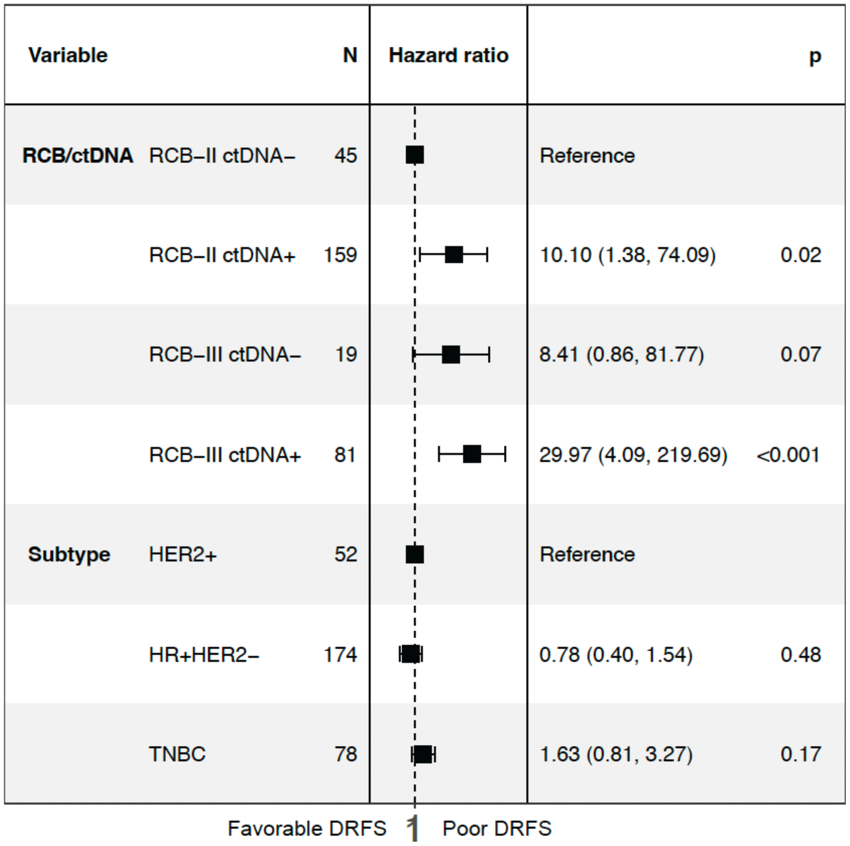

Pairwise Cox Regressions

Reference	Comparison	HzR	Lower 95 CI	Upper 95 CI	p value
RCB-II ctDNA+	RCB-II ctDNA-	0.10	0.01	0.73	0.023
RCB-II ctDNA+	RCB-III ctDNA+	2.97	1.86	4.74	<0.001
RCB-II ctDNA+	RCB-III ctDNA-	0.83	0.25	2.77	0.764
RCB-II ctDNA-	RCB-III ctDNA+	29.97	4.09	219.69	0.001
RCB-II ctDNA-	RCB-III ctDNA-	8.40	0.86	81.67	0.067
RCB-III ctDNA+	RCB-III ctDNA-	0.28	0.09	0.92	0.036

B ctDNA status at T3 and RCB-II/II

Pairwise log-rank tests

	RCB-II ctDNA+	RCB-II ctDNA-	RCB-III ctDNA+
RCB-II ctDNA-	<0.001	NA	NA
RCB-III ctDNA+	0.008	<0.001	NA
RCB-III ctDNA-	0.025	0.113	<0.001

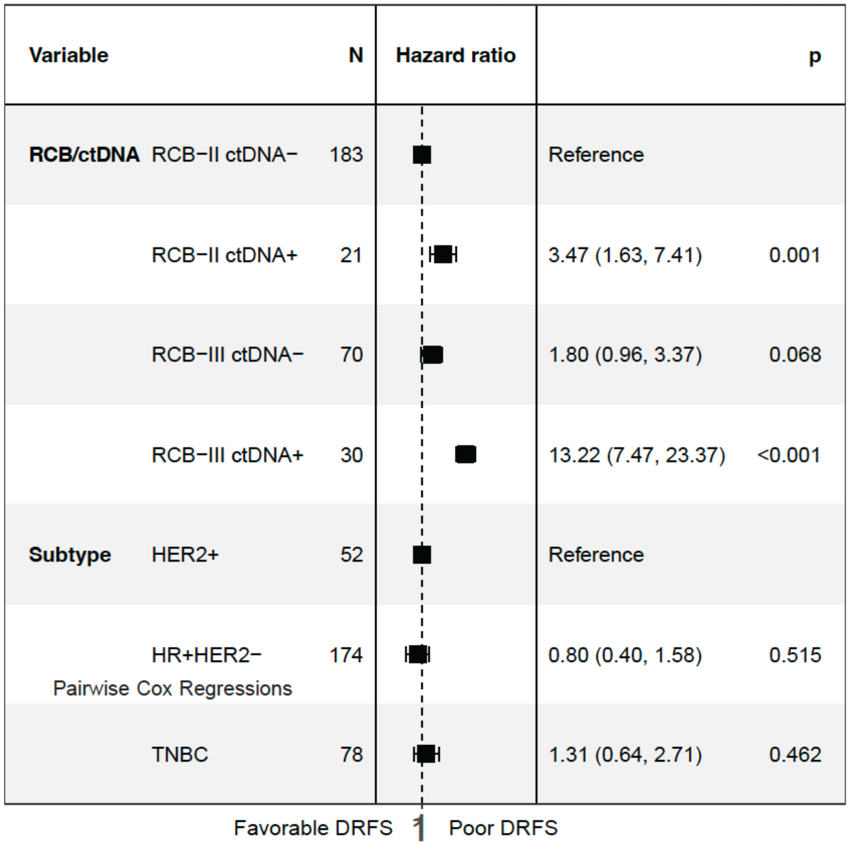

Pairwise Cox Regressions

Reference	Comparison	HzR	Lower 95 CI	Upper 95 CI	p value
RCB-II ctDNA+	RCB-II ctDNA-	0.29	0.13	0.61	0.001
RCB-II ctDNA+	RCB-III ctDNA+	3.80	1.78	8.14	0.001
RCB-II ctDNA+	RCB-III ctDNA-	0.52	0.22	1.20	0.123
RCB-II ctDNA-	RCB-III ctDNA+	13.23	7.48	23.39	0.000
RCB-II ctDNA-	RCB-III ctDNA-	1.80	0.96	3.37	0.068
RCB-III ctDNA+	RCB-III ctDNA-	0.14	0.07	0.26	<0.001

Figure S2. Circulating tumor DNA (ctDNA) status and risk stratification in patients with neoadjuvant therapy (NAT)-resistant tumors defined as residual cancer burden (RCB)-II or RCB-III. Patients with residual cancer RCB-II or RCB-III were stratified by **A.** ctDNA status at pretreatment (T0, **left panels**) or **B.** post-NAT before surgery (T3, **right panels**). **(top)** Table showing the results of pairwise Kaplan-Meier analyses with p-values from log-rank tests. **(middle)** Forest plots from Cox regression analyses showing estimates of hazard ratios and 95% confidence intervals (CI) adjusted for hormone receptor subtype. The p-values were calculated using the Wald test. **(bottom)** Table showing the results of pairwise Cox regression analyses showing estimates of hazard ratios (HzR) and 95% confidence intervals (CI) adjusted for hormone receptor subtype.

ctDNA dynamics in RCB-II/RCB-III

A

T1 (week 3) T2 (week 12) T3 (post-NAT)

Pairwise log-rank tests

	Persistent negative	Cleared at T1	Cleared at T2	Cleared at T3
Cleared at T1	0.135	NA	NA	NA
Cleared at T2	0.135	0.945	NA	NA
Cleared at T3	<0.001	0.002	0.002	NA
No clearance at T3	<0.001	<0.001	<0.001	0.001

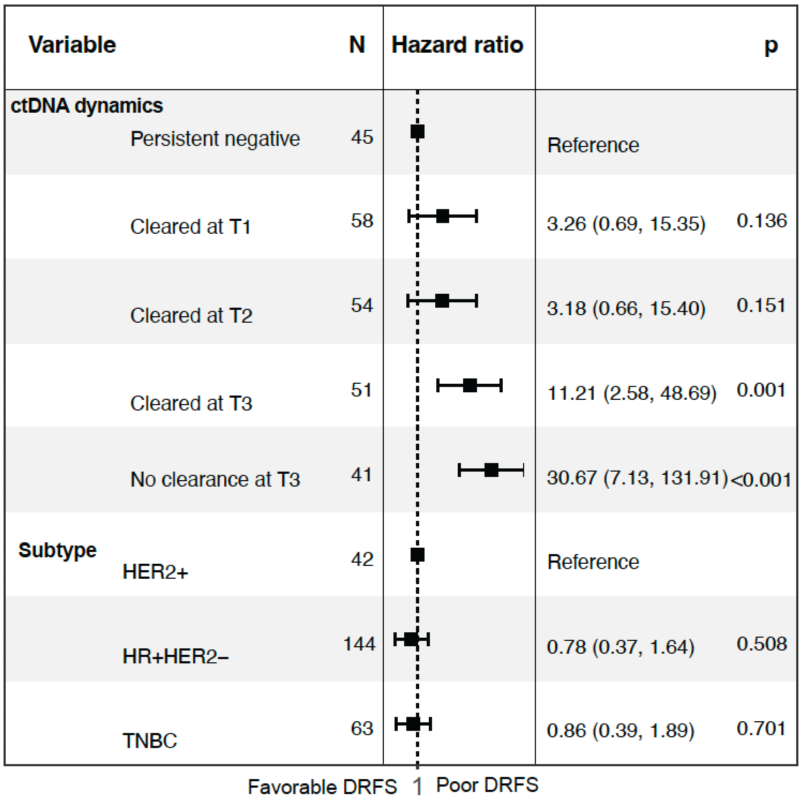

Pairwise Cox regression

Reference	Comparison	HzR	Lower 95 CI	Upper 95 CI	p value
Persistent negative	Cleared at T1	3.26	0.69	15.35	0.136
Persistent negative	Cleared at T2	3.18	0.66	15.40	0.151
Persistent negative	Cleared at T3	11.21	2.58	48.69	0.001
Persistent negative	No clearance at T3	30.67	7.13	131.91	<0.001
Cleared at T1	Cleared at T2	0.98	0.35	2.70	0.962
Cleared at T1	Cleared at T3	3.44	1.49	7.94	0.004
Cleared at T1	No clearance at T3	9.42	4.17	21.26	<0.001
Cleared at T2	Cleared at T3	3.53	1.48	8.39	0.004
Cleared at T2	No clearance at T3	9.65	4.17	22.36	<0.001
Cleared at T3	No clearance at T3	2.74	1.54	4.86	0.001

B

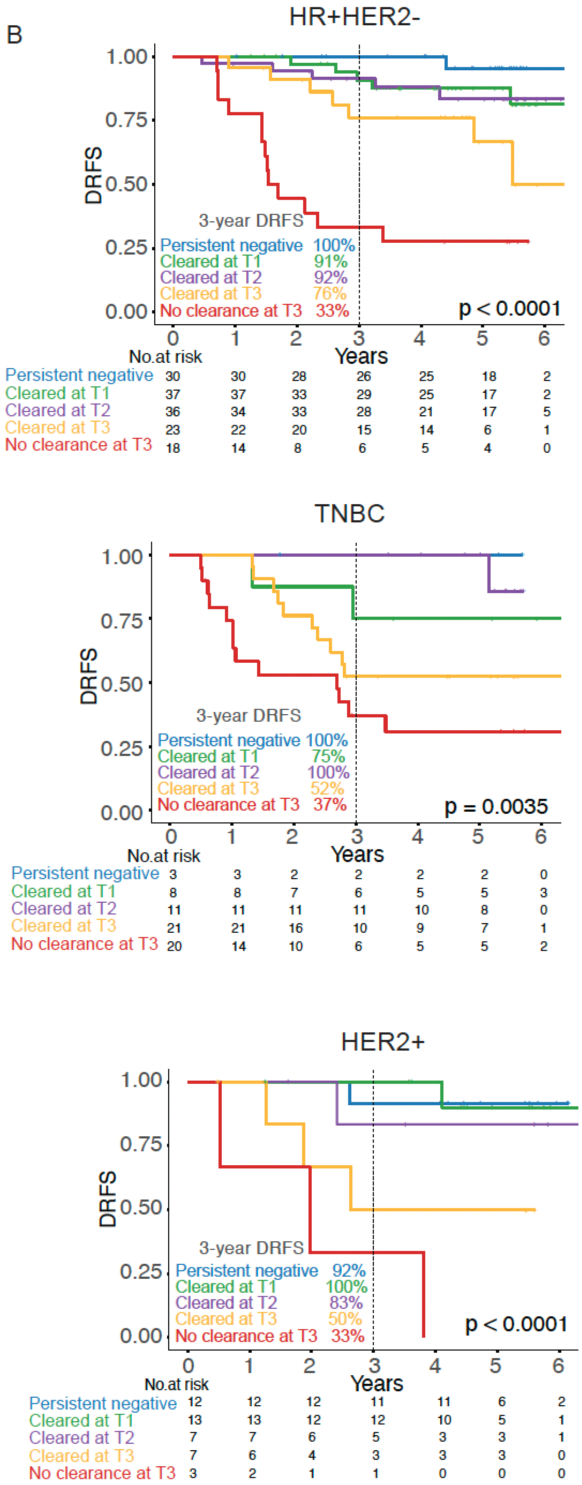

Figure S3. Risk stratification using circulating tumor DNA (ctDNA) dynamics (timing of ctDNA clearance) during neoadjuvant therapy (NAT) in patients with NAT-resistant tumors. Patients with NAT-resistant tumors defined as moderate (RCB-II) or extensive (RCB-III) residual cancer were stratified by ctDNA dynamics: persistent ctDNA-negative, ctDNA cleared at T1 (week 3), T2 (week 12), or T3 (post-NAT before surgery), and no ctDNA clearance post-NAT before surgery (T3). The prognostic value of ctDNA dynamics for predicting distant recurrence-free survival (DRFS) was examined. **A.** (**top**) Table showing the results of pairwise Kaplan-Meier analyses with p-values from log-rank tests. (**middle**) Forest plots from Cox regression analyses showing estimates of hazard ratios (HzR) and 95% confidence intervals (CI) adjusted for receptor subtype. The p-values were calculated using the Wald test. (**bottom**) Table showing the results of pairwise Cox regression analyses

showing estimates of HzR and 95% CI adjusted for receptor subtype. **B.** Kaplan-Meier estimates for 3-year DRFS rates in patients with RCB-II/III stratified by ctDNA dynamics across receptor subtypes: (**left**) hormone receptor-positive/HER2-negative (HR+HER2-), (**middle**) triple-negative breast cancer (TNBC), and (**right**) HER2-positive (HER2+).

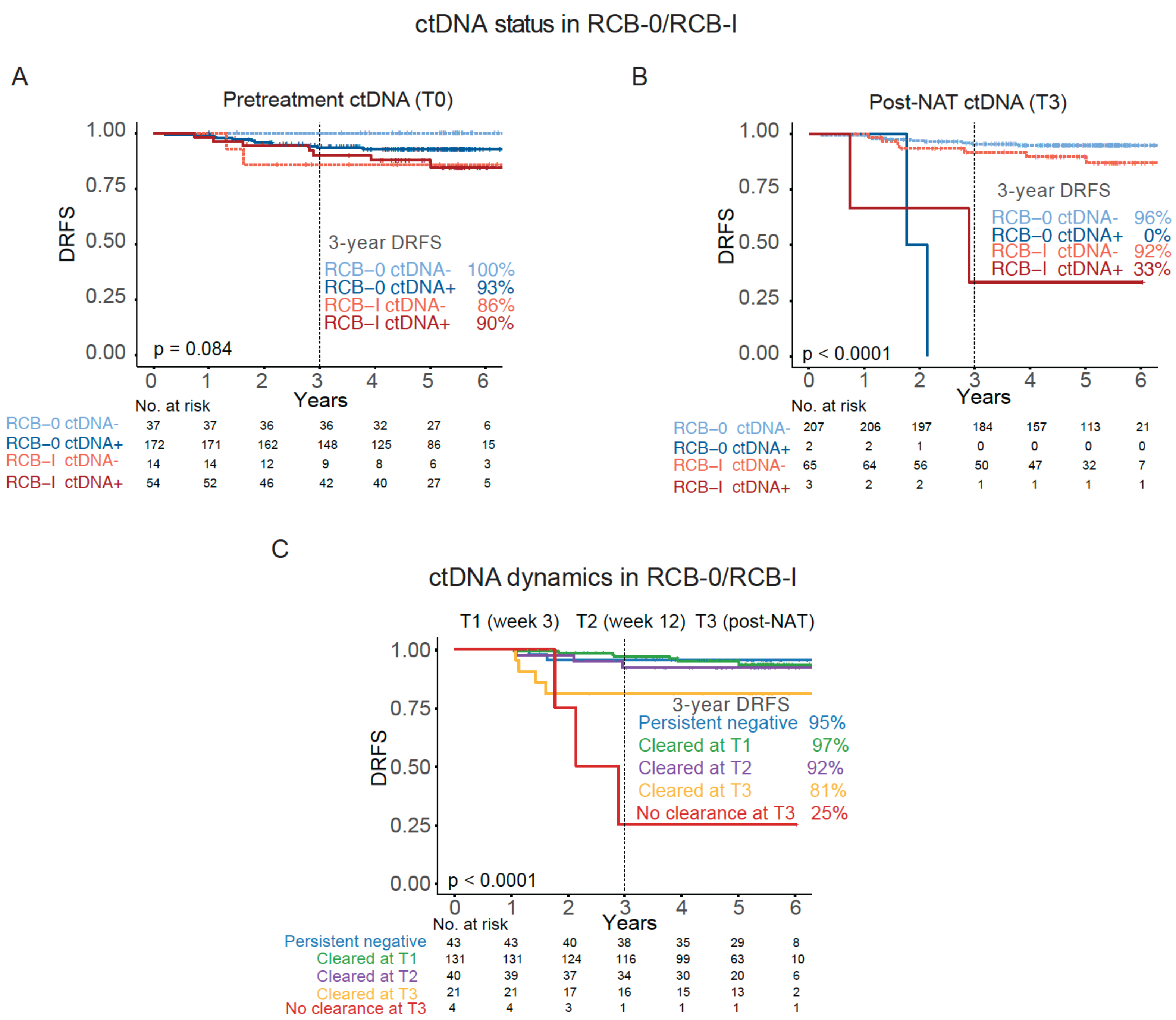
ctDNA status in RCB-0/RCB-I
A
Pretreatment ctDNA (T0)
DRFS
1.00
0.75
0.50
0.25
0.00
p = 0.084
3-year DRFS
RCB–0 ctDNA- 100%
RCB–0 ctDNA+ 93%
RCB–I ctDNA- 86%
RCB–I ctDNA+ 90%
0 1 2 3 4 5 6
Years
No. at risk
RCB–0 ctDNA- 37 37 36 36 32 27 6
RCB–0 ctDNA+ 172 171 162 148 125 86 15
RCB–I ctDNA- 14 14 12 9 8 6 3
RCB–I ctDNA+ 54 52 46 42 40 27 5
B
Post-NAT ctDNA (T3)
DRFS
1.00
0.75
0.50
0.25
0.00
p < 0.0001
3-year DRFS
RCB–0 ctDNA- 96%
RCB–0 ctDNA+ 0%
RCB–I ctDNA- 92%
RCB–I ctDNA+ 33%
0 1 2 3 4 5 6
Years
No. at risk
RCB–0 ctDNA- 207 206 197 184 157 113 21
RCB–0 ctDNA+ 2 2 1 0 0 0 0
RCB–I ctDNA- 65 64 56 50 47 32 7
RCB–I ctDNA+ 3 2 2 1 1 1 1
C
ctDNA dynamics in RCB-0/RCB-I
T1 (week 3) T2 (week 12) T3 (post-NAT)
DRFS
1.00
0.75
0.50
0.25
0.00
p < 0.0001
3-year DRFS
Persistent negative 95%
Cleared at T1 97%
Cleared at T2 92%
Cleared at T3 81%
No clearance at T3 25%
0 1 2 3 4 5 6
Years
No. at risk
Persistent negative 43 43 40 38 35 29 8
Cleared at T1 131 131 124 116 99 63 10
Cleared at T2 40 39 37 34 30 20 6
Cleared at T3 21 21 17 16 15 13 2
No clearance at T3 4 4 3 1 1 1 1

Figure S4. Risk stratification of breast tumors responsive to neoadjuvant therapy (NAT) using circulating tumor DNA (ctDNA). Kaplan-Meier estimates for 3-year distant recurrence-free survival (DRFS) rates in patients with NAT-responsive tumors defined as residual cancer burden, RCB-0 (pathologic complete response) or RCB-I (limited RCB) stratified by **A.** ctDNA status at pretreatment (T0) or **B.** ctDNA status post-NAT before surgery (T3); and **C.** in patients with RCB-0/RCB-I stratified by ctDNA dynamics (timing of ctDNA clearance): persistent ctDNA-negative, ctDNA cleared at T1 (week 3), T2 (week 12), or T3 (post-NAT before surgery), and no ctDNA clearance post-NAT before surgery. The p-values were calculated using the log-rank test.

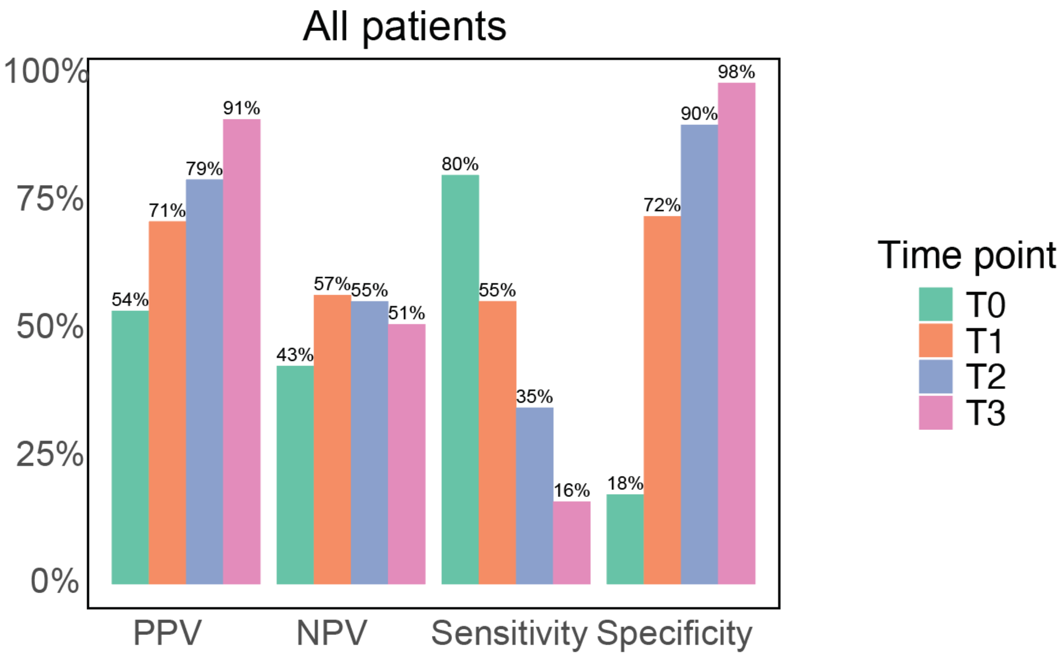

Figure S5. Performance metrics of the ctDNA assay for predicting residual cancer burden (RCB) status (RCB-0/I versus RCB-II/III) across receptor subtypes and time points during neoadjuvant therapy (NAT). The positive predictive value (PPV) and sensitivity for predicting moderate or extensive RCB (RCB-II/III), the negative predictive value (NPV) and specificity for predicting pathologic complete response or limited RCB (RCB-0/I) were calculated in all patients. ctDNA testing was performed at pretreatment (T0), weeks 3 (T1) and 12 (T2) after treatment initiation, and post-NAT before surgery (T3).

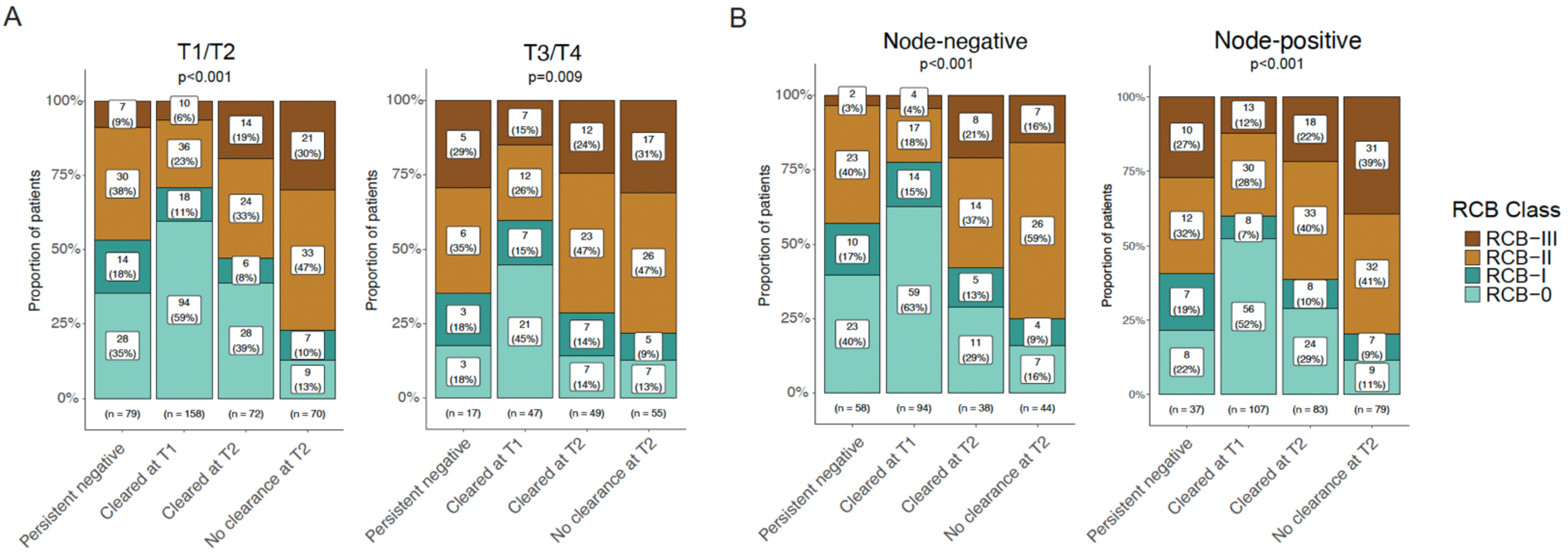

Figure S6. The association between early circulating tumor DNA (ctDNA) dynamics and residual cancer burden (RCB) class by clinical T and N stages. Patients were grouped by early ctDNA dynamics: persistent negative, cleared at T1 (week 3) or T2 (week 12), and no clearance at T2 and stratified by **A**. clinical T stage (T1/T2 and T3/T4), and **B.** clinical N stage (node-positive and node-negative). Bar plots showing the proportion of RCB classes in each early ctDNA dynamics group. The percentages may not add up to 100% due to rounding. The p-values were calculated using the Chi-squared test.

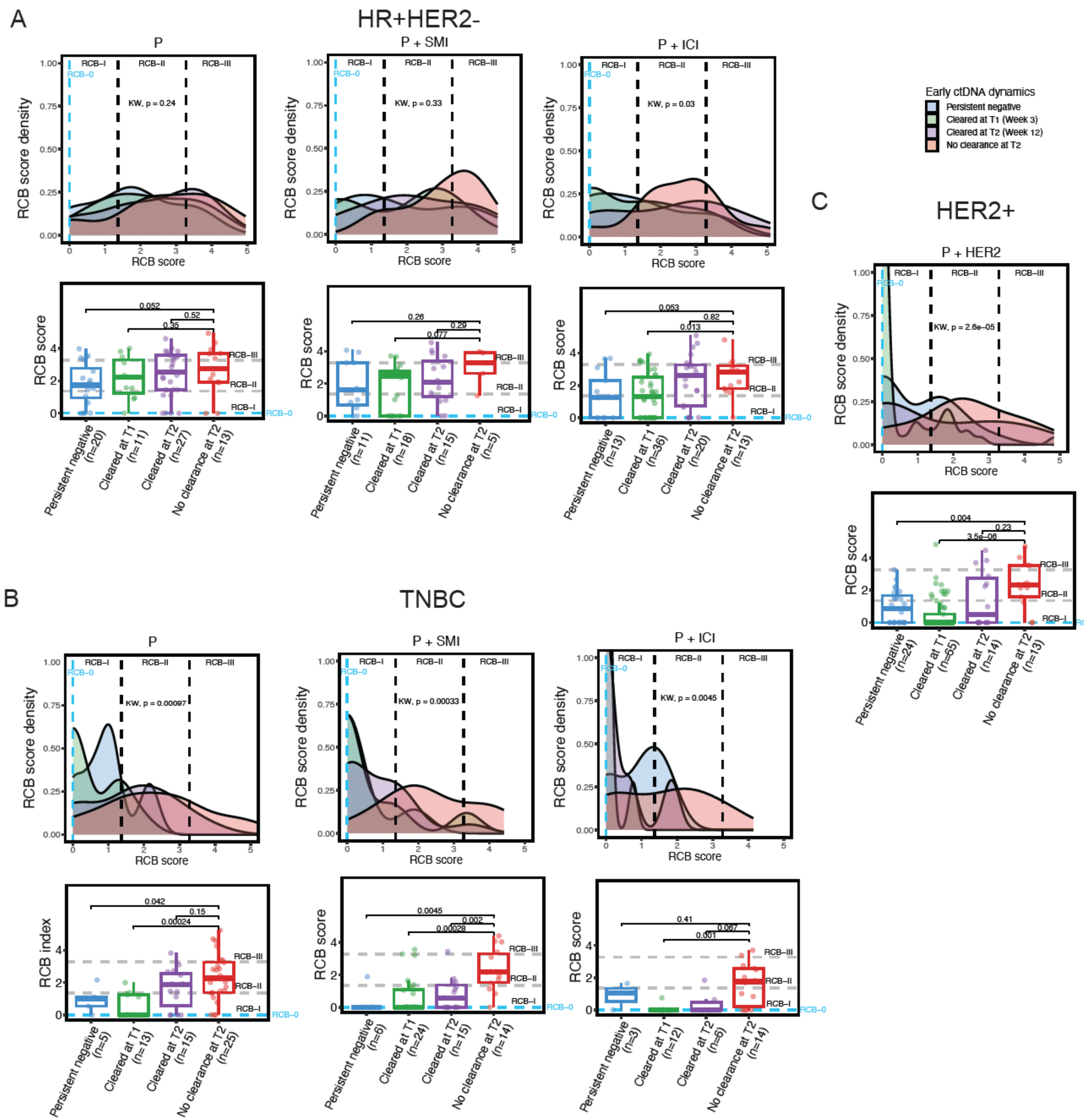
A
HR+HER2-
P
P + SMI
P + ICI
KW, p = 0.24
KW, p = 0.33
KW, p = 0.03
RCB score density
RCB score
RCB–0
RCB–I
RCB–II
RCB–III
Early ctDNA dynamics
Persistent negative
Cleared at T1 (Week 3)
Cleared at T2 (Week 12)
No clearance at T2
Persistent negative (n=20)
Cleared at T1 (n=11)
Cleared at T2 (n=27)
No clearance at T2 (n=13)
Persistent negative (n=11)
Cleared at T1 (n=18)
Cleared at T2 (n=15)
No clearance at T2 (n=5)
Persistent negative (n=13)
Cleared at T1 (n=36)
Cleared at T2 (n=20)
No clearance at T2 (n=13)
0.052
0.52
0.35
0.26
0.29
0.077
0.053
0.82
0.013
B
TNBC
P
P + SMI
P + ICI
KW, p = 0.00097
KW, p = 0.00033
KW, p = 0.0045
RCB index
0.042
0.15
0.00024
0.0045
0.002
0.00028
0.41
0.067
0.001
Persistent negative (n=5)
Cleared at T1 (n=13)
Cleared at T2 (n=15)
No clearance at T2 (n=25)
Persistent negative (n=6)
Cleared at T1 (n=24)
Cleared at T2 (n=15)
No clearance at T2 (n=14)
Persistent negative (n=3)
Cleared at T1 (n=12)
Cleared at T2 (n=6)
No clearance at T2 (n=14)
C
HER2+
P + HER2
KW, p = 2.6e–05
0.004
0.23
3.5e–06
Persistent negative (n=24)
Cleared at T1 (n=65)
Cleared at T2 (n=14)
No clearance at T2 (n=13)

Figure S7. The impact of early circulating tumor DNA (ctDNA) dynamics on residual cancer burden (RCB) score distribution by the type of treatment received. Patients were stratified by receptor subtypes: **A.** hormone receptor (HR)-positive/HER2-negative (HR+HER2-), **B.** triple-negative breast cancer (TNBC), and **C.** HER2-positive (HER2+) and the type of treatment received, including paclitaxel (P) alone, P + a small molecule inhibitor (P + SMI), P plus an immune checkpoint inhibitor (P + ICI), or P plus HER2-targeted drug (P + HER2). In each treatment type, patients were grouped by early ctDNA dynamics: persistent negative, cleared at T1 (week 3) or T2 (week 12), and no clearance at T2. The distribution of RCB scores for each group was visualized using density (upper panels) and box-and-whisker (lower panels) plots. The density plots visualize the distribution of continuous values (RCB scores), with peaks showing where the values are concentrated. The total area under each distribution curve is equal to 1. The box-and-whisker plot shows the interquartile range (IQR) of the RCB scores for a given group divided into quartiles, with Q1 (the lower end of the box), Q2 (the median), and Q3 (the upper end of the box). The whiskers from the box represent the data outside the upper and lower quartiles. The p-values for multi-group comparisons (>2) were calculated using the Kruskal-Wallis (KW) and pairwise comparisons using the Wilcoxon rank-sum test with adjustment for multiple hypotheses testing using the Bonferroni correction.

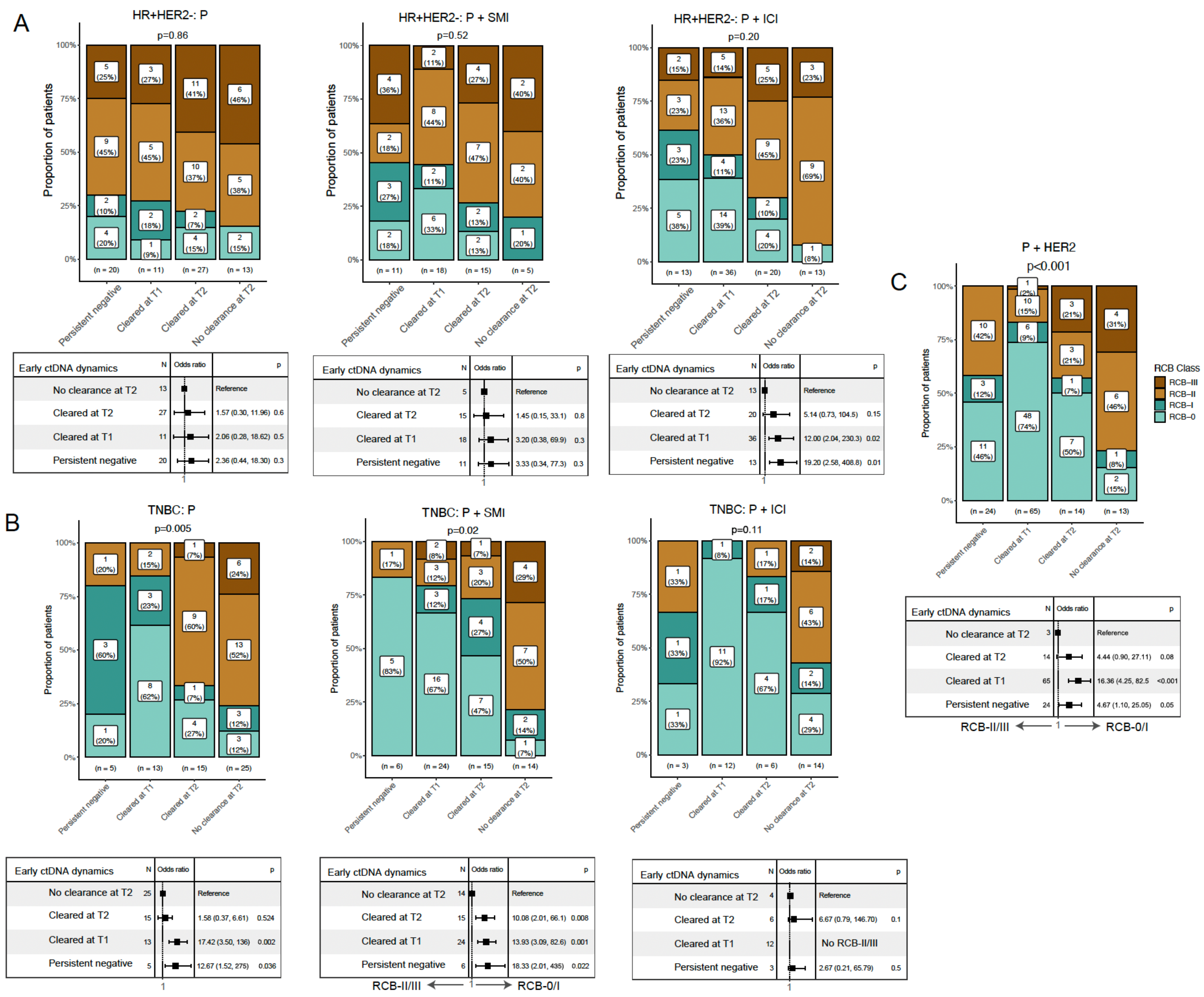
A
HR+HER2-: P
p=0.86
HR+HER2-: P + SMI
p=0.52
HR+HER2-: P + ICI
p=0.20
Proportion of patients
Persistent negative
Cleared at T1
Cleared at T2
No clearance at T2
Early ctDNA dynamics | N | Odds ratio | p
No clearance at T2 13 Reference
Cleared at T2 27 1.57 (0.30, 11.96) 0.6
Cleared at T1 11 2.06 (0.28, 18.62) 0.5
Persistent negative 20 2.36 (0.44, 18.30) 0.3
No clearance at T2 5 Reference
Cleared at T2 15 1.45 (0.15, 33.1) 0.8
Cleared at T1 18 3.20 (0.38, 69.9) 0.3
Persistent negative 11 3.33 (0.34, 77.3) 0.3
No clearance at T2 13 Reference
Cleared at T2 20 5.14 (0.73, 104.5) 0.15
Cleared at T1 36 12.00 (2.04, 230.3) 0.02
Persistent negative 13 19.20 (2.58, 408.8) 0.01
B
TNBC: P
p=0.005
TNBC: P + SMI
p=0.02
TNBC: P + ICI
p=0.11
No clearance at T2 25 Reference
Cleared at T2 15 1.58 (0.37, 6.61) 0.524
Cleared at T1 13 17.42 (3.50, 136) 0.002
Persistent negative 5 12.67 (1.52, 275) 0.036
No clearance at T2 14 Reference
Cleared at T2 15 10.08 (2.01, 66.1) 0.008
Cleared at T1 24 13.93 (3.09, 82.6) 0.001
Persistent negative 6 18.33 (2.01, 435) 0.022
No clearance at T2 4 Reference
Cleared at T2 6 6.67 (0.79, 146.70) 0.1
Cleared at T1 12 No RCB-II/III
Persistent negative 3 2.67 (0.21, 65.79) 0.5
RCB-II/III ← 1 → RCB-0/I
C
P + HER2
p<0.001
RCB Class
RCB–III
RCB–II
RCB–I
RCB–0
No clearance at T2 3 Reference
Cleared at T2 14 4.44 (0.90, 27.11) 0.08
Cleared at T1 65 16.36 (4.25, 82.5 <0.001
Persistent negative 24 4.67 (1.10, 25.05) 0.05
RCB-II/III ← 1 → RCB-0/I

Figure S8. The association between early circulating tumor DNA (ctDNA) dynamics and residual cancer burden (RCB) class by the type of treatment received. Patients were stratified by receptor subtypes: **A.** hormone receptor (HR)-positive/HER2-negative (HR+HER2-), **B.** triple-negative breast cancer (TNBC), and **C.** HER2-positive (HER2+) and the type of treatment received, including paclitaxel (P) alone, P + a small molecule inhibitor (P + SMI), P plus an immune checkpoint inhibitor (P + ICI), or P plus HER2-targeted drug (P + HER2). In each treatment type, patients were grouped by early ctDNA dynamics: persistent negative, cleared at T1 (week 3) or T2 (week 12), and no clearance at T2. The association between early ctDNA dynamics and RCB class is shown in the bar (upper panels) and forest (lower panels) plots. Bar plots show the RCB class proportion in each early ctDNA dynamics group. The p-values were calculated using the Chi-squared test. The forest plots from logistic regression analyses show the odds ratio estimates and 95% confidence intervals. The p-values were calculated from likelihood ratio tests.

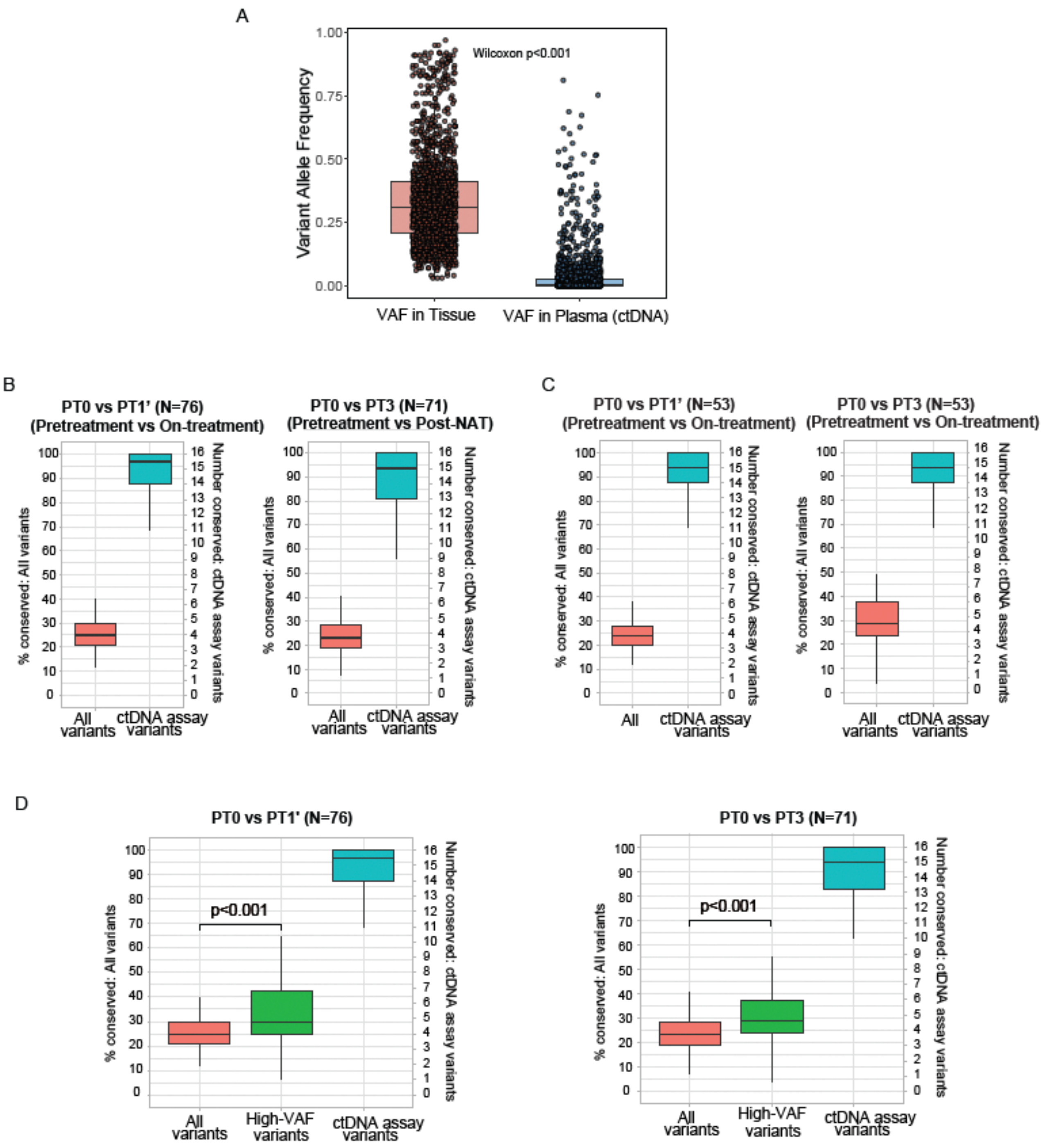

Figure S9. Serial tumor mutation profiling of neoadjuvant therapy (NAT)-resistant tumors reveals the high conservation of patient-specific circulating tumor DNA (ctDNA) assay variants in the tissue over time. Mutation profiling by whole exome sequencing of matched tumor tissue samples was performed at pretreatment (PT0), on-treatment (week 3 or week 12, PT1'), and post-NAT at surgery (PT3) for a subset of patients with residual cancer burden (RCB)-II or RCB-III. **A**. Box plots comparing the distribution of the variant allele frequencies (VAF) of the patient-specific ctDNA assays in the tumor tissue versus those in the plasma (ctDNA) at pretreatment (T0). The p-value was calculated using the Wilcoxon signed-rank test. **B.** Paired mutation profiling data were available for pretreatment (PT0) and on-treatment (PT1) tumors (n=76, **left**) and pretreatment (PT0) and post-NAT (PT3) tumors (n=71, **right**). The orange box plots show the distribution of the percentages of conserved somatic variants detected in paired tumor samples collected at PT0 and PT1, or at PT0 and PT3. The aqua box plots show the distribution of the percentages (left y-axis) or the number (right y-

axis, up to 16 for each patient) of conserved personalized ctDNA assay variants detected in paired tumor samples collected at PT0 and PT1, and at PT0 and PT3. The same analysis (variant conservation) was restricted to **C**. patients with serial tumor samples for all three time points (n=53, 56%), and **D.** to mutations with high VAF (> median VAF) (green boxes). The p-value was calculated using the Wilcoxon rank sum test.

Table S1. Patients and clinicopathologic characteristics. Patients were grouped by receptor subtypes: hormone receptor-positive/HER2-negative (HR+HER2-), triple-negative breast cancer (TNBC), and HER2-positive breast cancer. The survival endpoint of the study is distant recurrence-free survival (DRFS). For categorical variables, the proportions were compared using Fisher's exact test. For continuous variables (age at screening), the distributions were compared using a t-test.

.

**Clinicopathologic variable**		**HR+HER2-**	**TNBC**	**HER2+**	**Total**	**p**
		n (%)	n (%)	n (%)	n (%)	
Clinical T Stage						0.121
	T1	7 (2.4)	11 (4.7)	7 (3.8)	25 (3.5)	
	T2	183 (62.7)	158 (67.8)	130 (70.3)	471 (66.3)	
	T3	90 (30.8)	53 (22.7)	37 (20.0)	180 (25.4)	
	T4	12 (4.1)	11 (4.7)	11 (5.9)	34 (4.8)	
Clinical N Stage						<0.001
	Node-negative	96 (33.0)	123 (53.5)	82 (45.3)	301 (42.9)	
	Node-positive	195 (67.0)	107 (46.5)	99 (54.7)	401 (57.1)	
Grade						<0.001
	1/2	101 (43.2)	14 (8.4)	38 (30.6)	153 (29.2)	
	3	133 (56.8)	152 (91.6)	86 (69.4)	371 (70.8)	
MammaPrint						<0.001
	High 1	193 (64.3)	24 (10.1)	111 (59.7)	328 (45.4)	
	High 2	107 (35.7)	213 (89.9)	75 (40.3)	395 (54.6)	
Residual Cancer Burder (RCB)						<0.001
	RCB-0	55 (18.6)	87 (37.7)	99 (54.7)	241 (34.0)	
	RCB-I	30 (10.1)	35 (15.2)	15 (8.3)	80 (11.3)	
	RCB-II	130 (43.9)	75 (32.5)	50 (27.6)	255 (36.0)	
	RCB-III	81 (27.4)	34 (14.7)	17 (9.4)	132 (18.6)	
Age						0.758
	Mean (SD)	48.9 (11.0)	48.5 (11.9)	49.3 (9.7)	48.9 (11.0)	

Table S2. Univariate Cox regression analysis. Correlation of clinicopathological variables with distant recurrence-free survival (DRFS). Abbreviations: CI – confidence interval; HR – hormone receptor; RCB – residual cancer burden; TNBC – triple-negative breast cancer.

Variable*	Factor	Reference	Hazard ratio	Lower 95% CI	Upper 95% CI	Wald p
**ctDNA**	ctDNA+ at T0	ctDNA-	5.50	2.43	12.49	<0.0001
	ctDNA+ at T3		10.36	6.92	15.50	<0.0001
**Clinical T stage**	T3/T4	T1/T2	2.17	1.53	3.07	<0.0001
**Clinical N stage**	Node+	Node-	1.47	1.02	2.12	0.0373
Grade	3	1/2	1.31	0.82	2.07	0.2557
MammaPrint	High 2	High 1	1.25	0.88	1.76	0.2066
**Receptor Subtype**	HR+HER2-	HER2+	0.53	0.32	0.88	0.0143
	TNBC		1.22	0.84	1.76	0.2944
**RCB class**	RCB-I	RCB-0	1.96	0.89	4.33	0.0939
	RCB-II		3.20	1.82	5.62	0.0001
	RCB-III		7.63	4.35	13.37	<0.0001

Variable*	Factor	Reference	Hazard ratio	Lower 95% CI	Upper 95% CI	Wald p
**ctDNA dynamics**	Cleared at T1	Persistent ctDNA-	1.92	0.64	5.75	0.2428
	Cleared at T2		2.47	0.78	7.88	0.1260
	Cleared at T3		8.96	3.11	25.85	<0.0001
	No clearance at T3		30.00	10.60	84.87	<0.0001
**Clinical T stage**	T3/T4	T1/T2	2.03	1.33	3.11	0.0011
**Clinical N stage**	Node+	Node-	1.87	1.17	2.96	0.0082
Grade	3	1/2	1.11	0.65	1.90	0.6906
MammaPrint	high 2	high 1	1.27	0.83	1.94	0.2702
Receptor Subtype	HR+HER2-	HER2+	0.61	0.33	1.12	0.1090
	TNBC		1.28	0.81	2.03	0.2872
**RCB class**	RCB-I	RCB-0	2.29	0.92	5.70	0.0741
	RCB-II		3.30	1.66	6.56	0.0007
	RCB-III		9.15	4.64	18.02	<0.0001

*Variables in bold are considered statistically significant

Table S3. Types of treatments in I-SPY2. Patients were assigned to different arms in the I-SPY2 trial. The control arm and the treatment arms, for which ctDNA analysis has been completed for all evaluable patients, were included in the analysis. These arms were grouped into four treatment types (see **Methods**).

Treatment Type	N by Agent	N by Subtype	Total
Control	Paclitaxel (N=161)	HR+HER2- (n=88)<br>TNBC (n=73)	161
Small molecule inhibitor-containing	Irinotecan + Talazoparib (n=49)<br>ABT 888 + Carboplatin (n=58)<br>MK-2206 (n=39)	HR+HER2- (n=66)<br>TNBC (n=80)	146
ICI-containing	Anti-PD-1 4 cycles (n=46)<br>Anti-PD-1 8 cycles (n=56)<br>Durvalumab + Olaparib (n=52)	HR+HER2- (n=105)<br>TNBC (n=49)	154
HER2-targeted	Pertuzumab + Trastuzumab (n=111)<br>Trastuzumab (n=8)<br>T-DM1 + Pertuzumab (n=42)	HER2+ (n=161)	161

Table S4. Conservation of patient-specific circulating tumor DNA (ctDNA) assay variants in serial tumors of patients stratified by ctDNA dynamics. Mutation profiling by whole exome sequencing of matched tumor tissue samples was performed at pretreatment (PT0), on-treatment (week 3 or week 12, PT1'), and post-NAT at surgery (PT3) for a subset of patients with residual cancer burden (RCB)-II or RCB-III**.** Patients were grouped by ctDNA dynamics: persistent ctDNA-negative, ctDNA cleared at T1 (week 3), T2 (week 12), or T3 (post-NAT before surgery), or no ctDNA clearance post-NAT before surgery. Tables showing the median, quartiles, and interquartile ranges of percent conservation of all variants and patient-specific ctDNA assay variants.

Paired WES data of matched tumor from PT0 and PT1' (N=42)

% conservation of all variants					% conservation of personalized ctDNA assay variants				
ctDNA dynamics	Median	q25	q75	IQR	ctDNA dynamics	Median	q25	q75	IQR
Persistent negative	21.5%	19.3%	25.0%	5.7%	Persistent negative	90.6%	85.9%	93.8%	7.8%
Cleared at T1	28.6%	25.6%	30.4%	4.7%	Cleared at T1	96.9%	79.7%	100.0%	20.3%
Cleared at T2	22.0%	19.6%	28.2%	8.6%	Cleared at T2	90.6%	84.4%	100.0%	15.6%
Cleared at T3	25.5%	19.4%	28.7%	9.3%	Cleared at T3	100.0%	95.3%	100.0%	4.7%
No clearance	24.7%	23.6%	27.7%	4.1%	No clearance	100.0%	100.0%	100.0%	0.0%

Paired WES data of matched tumor from PT0 and PT3 (N=41)

% conservation of all variants					% conservation of personalized ctDNA assay variants				
ctDNA dynamics	Median	q25	q75	IQR	ctDNA dynamics	Median	q25	q75	IQR
Persistent negative	23.2%	21.4%	25.9%	4.5%	Persistent negative	87.5%	87.5%	93.8%	6.3%
Cleared at T1	22.9%	18.0%	30.2%	12.2%	Cleared at T1	81.3%	56.3%	95.3%	39.1%
Cleared at T2	18.1%	15.0%	22.0%	7.0%	Cleared at T2	93.8%	85.9%	100.0%	14.1%
Cleared at T3	20.0%	15.5%	25.3%	9.8%	Cleared at T3	93.8%	90.6%	100.0%	9.4%
No clearance	23.2%	22.2%	27.1%	4.9%	No clearance	100.0%	90.6%	100.0%	9.4%

q=quartile, IQR=interquatile
