# Supplementary material for: Circulating tumor DNA refines risk stratification of neoadjuvant therapy-resistant breast tumors
Source: Nat Commun. 2025 Dec 9;16:9945. doi: 10.1038/s41467-025-65432-5 (PMC12690079; doi:10.1038/s41467-025-65432-5)
Supplement: Supplementary file 2 — Reporting Summary [file 41467_2025_65432_MOESM2_ESM.pdf]

nature portfolio

Corresponding author(s): Mark Jesus M. Magbanua
mark.magbanua@ucsf.edu

Last updated by author(s): Apr 11, 2025

Reporting Summary

Nature Portfolio wishes to improve the reproducibility of the work that we publish. This form provides structure for consistency and transparency in reporting. For further information on Nature Portfolio policies, see our Editorial Policies and the Editorial Policy Checklist.

Statistics

For all statistical analyses, confirm that the following items are present in the figure legend, table legend, main text, or Methods section.

n/a	Confirmed	
☐	☒	The exact sample size (n) for each experimental group/condition, given as a discrete number and unit of measurement
☐	☒	A statement on whether measurements were taken from distinct samples or whether the same sample was measured repeatedly
☒	☐	The statistical test(s) used AND whether they are one- or two-sided<br>*Only common tests should be described solely by name; describe more complex techniques in the Methods section.*
☐	☒	A description of all covariates tested
☐	☒	A description of any assumptions or corrections, such as tests of normality and adjustment for multiple comparisons
☐	☒	A full description of the statistical parameters including central tendency (e.g. means) or other basic estimates (e.g. regression coefficient) AND variation (e.g. standard deviation) or associated estimates of uncertainty (e.g. confidence intervals)
☐	☒	For null hypothesis testing, the test statistic (e.g. F, t, r) with confidence intervals, effect sizes, degrees of freedom and P value noted<br>*Give P values as exact values whenever suitable.*
☒	☐	For Bayesian analysis, information on the choice of priors and Markov chain Monte Carlo settings
☒	☐	For hierarchical and complex designs, identification of the appropriate level for tests and full reporting of outcomes
☒	☐	Estimates of effect sizes (e.g. Cohen's d, Pearson's r), indicating how they were calculated

Our web collection on statistics for biologists contains articles on many of the points above.

Software and code

Policy information about availability of computer code

Data collection	ctDNA data was generated by Natera, Inc. Whole exome sequencing of pretreatment tumors was performed using the Illumina platform (Illumina Inc, California, USA), followed by prioritization of somatic single nucleotide variants for design and application of personalized ctDNA assays .
Data analysis	Sequence data was aligned to human reference genome version GRCh38 using Burrows-Wheeler Alignment tool (version 0.7.12). Somatic mutations were detected for each tumor and PBL data pair using Varscan2, GATK MuTect2 and HaplotypeCaller (version 3.6-0-g89b7209), MuTect, and Vardict. Tumor mutation burden was calculated as the total number of nonsynonymous mutations detected in more than 2 out of 5 mutation callers for each sample.<br><br>Multiplex PCR primer pairs targeting 16 highly ranked tumor-specific variants were designed as previously described. Briefly, somatic small nucleotide variants (SNVs) present in the tumor but absent in the germline were used to select the 16 targets, with prioritization based upon multiple factors, including the observed variant allele frequency in the tumor tissue and the associated background noise profile in the plasma. This prioritized list of variants was then used to design PCR amplicons based on optimized design parameters, ensuring uniqueness in the human genome, amplicon efficiency, and primer interaction. Next, multiplexed targeted PCR was conducted followed by amplicon deep sequencing on an Illumina platform. A sample was considered ctDNA positive when ≥2 out of the 16 selected target mutations were present at above a predefined threshold. Variant allele frequencies were determined for each of the 16 target mutations. Absolute ctDNA levels (mean tumor [ctDNA] molecules per mL [MTM/mL]) in the plasma were determined by normalizing variant allele frequencies by the plasma volume used for each sample. At each time point, MTM/mL was calculated from all 16 tested targets (including undetected targets).<br><br>All statistical analyses were performed using R (R Foundation for Statistical Computing, Vienna, Austria; URL https://www.R-project.org/). R scripts are available upon request.

For manuscripts utilizing custom algorithms or software that are central to the research but not yet described in published literature, software must be made available to editors and reviewers. We strongly encourage code deposition in a community repository (e.g. GitHub). See the Nature Portfolio guidelines for submitting code & software for further information.

Data

Policy information about availability of data

All manuscripts must include a data availability statement. This statement should provide the following information, where applicable:

- Accession codes, unique identifiers, or web links for publicly available datasets
- A description of any restrictions on data availability
- For clinical datasets or third party data, please ensure that the statement adheres to our policy

De-identified subject-level data and/or clinical specimens are made available to members of the research community upon approval of the I-SPY Data Access and Publications Committee. Details of the application and review process are available at https://www.quantumleaphealth.org/for-investigators/clinicians-proposal-submissions/. I-SPY aims to make complete patient-level clinical datasets available for public access within 6 months of publication, as the data is complex and requires significant annotation to ensure its usability.

Research involving human participants, their data, or biological material

Policy information about studies with human participants or human data. See also policy information about sex, gender (identity/presentation), and sexual orientation and race, ethnicity and racism.

Reporting on sex and gender	The I-SPY2.2 trial in early breast cancer enrolls adult women.
Reporting on race, ethnicity, or other socially relevant groupings	Self-reported race and ethnicity of the study population are not reported in the manuscript.
Population characteristics	The population consists of patients with high-risk (MammaPrint high) early-stage breast cancer receiving NAT in the I-SPY2 trial. Of the 723 evaluable patients, 300 (41%) were HR-positive/HER2-negative, 237 (33%) were TN, and 186 (26%) were HER2-positive.
Recruitment	Patients in this study were recruited by I-SPY 2 TRIAL (NCT01042379; IND 105139). Participants are invited to screen for I-SPY2 at the invitation of local clinical personnel at the time of breast cancer diagnosis at open study sites across the US. Participants did not receive compensation for trial participation.
Ethics oversight	Institutional review board from all participating sites approved the I-SPY2 protocol. All patients signed informed consent to allow research on and use of their biospecimen samples.

Note that full information on the approval of the study protocol must also be provided in the manuscript.

Field-specific reporting

Please select the one below that is the best fit for your research. If you are not sure, read the appropriate sections before making your selection.

☒ Life sciences ☐ Behavioural & social sciences ☐ Ecological, evolutionary & environmental sciences

For a reference copy of the document with all sections, see nature.com/documents/nr-reporting-summary-flat.pdf

Life sciences study design

All studies must disclose on these points even when the disclosure is negative.

Sample size	The population consists of 723 patients with high-risk (MammaPrint high) early-stage breast cancer receiving NAT in the I-SPY2 trial.
Data exclusions	The manuscript presents a detailed diagram illustrating how and why certain data were excluded (Figure 1). Patients were excluded when whole exome sequencing was unsuccessful and upon withdrawal of consent.
Replication	Due to sample size and biospecimen availability, ctDNA measurements were performed once on each sample and were not repeated.
Randomization	The patient population were randomized to different treatment arms of I-SPY2. Randomization was performed at the clinical trial level.
Blinding	The ctDNA measurements were conducted by Natera in a manner that kept them blinded to clinical data including survival and response outcomes. All clinical outcome data were collected by individuals blinded to the ctDNA results.

Reporting for specific materials, systems and methods

We require information from authors about some types of materials, experimental systems and methods used in many studies. Here, indicate whether each material, system or method listed is relevant to your study. If you are not sure if a list item applies to your research, read the appropriate section before selecting a response.

Materials & experimental systems

n/a	Involved in the study
☒	☐ Antibodies
☒	☐ Eukaryotic cell lines
☒	☐ Palaeontology and archaeology
☒	☐ Animals and other organisms
☐	☒ Clinical data
☒	☐ Dual use research of concern
☒	☐ Plants

Methods

n/a	Involved in the study
☒	☐ ChIP-seq
☒	☐ Flow cytometry
☒	☐ MRI-based neuroimaging

Clinical data

Policy information about clinical studies

All manuscripts should comply with the ICMJE guidelines for publication of clinical research and a completed CONSORT checklist must be included with all submissions.

Clinical trial registration	I-SPY2 TRIAL (NCT01042379; IND 105139)
Study protocol	Protocols and related documents are described in I-SPY2 efficacy papers: Rugo, H.S. et al. Adaptive Randomization of Veliparib-Carboplatin Treatment in Breast Cancer. N Engl J Med, 2016. 375(1): p. 23-34.
Data collection	Clinical data were collected at each site and submitted to Quantum Leap Healthcare Collaborative, the I-SPY2 sponsor.
Outcomes	The response endpoint is residual cancer burden (RCB), which quantifies the extent of residual disease after treatment, assessed at the time of surgery. The survival endpoint is distant recurrence-free survival (DRFS), calculated as the time between treatment consent and the first distant recurrence or death to any cause. Patients without a DRFS event were censored at the time of last follow-up.

Plants

Seed stocks	*Report on the source of all seed stocks or other plant material used. If applicable, state the seed stock centre and catalogue number. If plant specimens were collected from the field, describe the collection location, date and sampling procedures.*
Novel plant genotypes	*Describe the methods by which all novel plant genotypes were produced. This includes those generated by transgenic approaches, gene editing, chemical/radiation-based mutagenesis and hybridization. For transgenic lines, describe the transformation method, the number of independent lines analyzed and the generation upon which experiments were performed. For gene-edited lines, describe the editor used, the endogenous sequence targeted for editing, the targeting guide RNA sequence (if applicable) and how the editor was applied.*
Authentication	*Describe any authentication procedures for each seed stock used or novel genotype generated. Describe any experiments used to assess the effect of a mutation and, where applicable, how potential secondary effects (e.g. second site T-DNA insertions, mosiacism, off-target gene editing) were examined.*
